# Detection of Human Bladder Epithelial Cancerous Cells with Atomic Force Microscopy and Machine Learning

**DOI:** 10.3390/cells14010014

**Published:** 2024-12-26

**Authors:** Mikhail Petrov, Nadezhda Makarova, Amir Monemian, Jean Pham, Małgorzata Lekka, Igor Sokolov

**Affiliations:** 1 Department of Mechanical Engineering, Tufts University, Medford, MA 02155, USA; mikhail.petrov@tufts.edu (M.P.); nadia.proximi@gmail.com (N.M.); 2 Cellens, Inc., 529 Main Street, Suite 1M6, Boston, MA 02129, USA; 3 Department of Biophysical Microstructures, Institute of Nuclear Physics PAN, PL-31342 Kraków, Poland; malgorzata.lekka@ifj.edu.pl; 4 Departments of Biomedical Engineering and Physics, Tufts University, Medford, MA 02155, USA

**Keywords:** imaging, nanomedicine, cancer, atomic force microscopy, ringing mode, artificial intelligence, machine learning

## Abstract

The development of noninvasive methods for bladder cancer identification remains a critical clinical need. Recent studies have shown that atomic force microscopy (AFM), combined with pattern recognition machine learning, can detect bladder cancer by analyzing cells extracted from urine. However, these promising findings were limited by a relatively small patient cohort, resulting in modest statistical significance. In this study, we corroborated the AFM technique’s capability to identify bladder cancer cells with high accuracy using a controlled model system of genetically purified human bladder epithelial cell lines, comparing cancerous cells with nonmalignant controls. By processing AFM adhesion maps through machine learning algorithms, following previously established methods, we achieved an area under the ROC curve (AUC) of 0.97, with 91% accuracy in cancer cell identification. Furthermore, we enhanced cancer detection by incorporating multiple imaging channels recorded with AFM operating in Ringing mode, achieving an AUC of 0.99 and 93% accuracy. These results demonstrated strong statistical significance (*p* < 0.0001) in this well-defined model system. While this controlled study does not capture the biological variation present in clinical settings, it provides independent support for AFM-based detection methods and establishes a rigorous technical foundation for further clinical development of AFM imaging-based methods for bladder cancer detection.

## 1. Introduction

Developing new methods of accurate and early cancer detection is key to improving survival and quality of life [1]. Bladder cancer is one of the most common cancers and causes of cancer-related deaths both in the USA and globally [2,3]. It is the second leading cause of death from urological cancers worldwide. The 5-year survival rate drops from 95% for patients with cancer detected at the early stage to 10% for those at the metastatic stage [4]. The gold standard for diagnosis includes cystoscopy and tumor resection for pathology examination. Because of a high (50–80%) recurrence rate, frequent costly and invasive cystoscopy exams are required to monitor patients for recurrence and/or progression to a more advanced stage (once every 3–6 months, which is the current practice for patients with non-muscle-invasive tumors (75% of newly diagnosed bladder cancers [5]). The requirement for frequent cystoscopy makes bladder cancer the most expensive cancer per patient to diagnose, monitor, and treat. Numerous global authorities recognized it as a major health issue incurring a significant burden on health care systems [6]. Cystoscopy also has a limited ability to detect occult microscopic cancer and tumors in atypical locations as well as those higher up in the urinary tract (upper tract urothelial cancer). Finally, cystoscopy is invasive, requires a lot of resources, and can have potential complications (e.g., it can be associated with post-procedure dysuria/pain/burning, urinary urgency, infections, hematuria, and perforations). Thus, the development of an accurate, effective, and noninvasive test to detect bladder cancer is significant.

Atomic force microscopy (AFM) has transformed our ability to visualize and analyze samples at the nanoscale, including biological cells, offering unique insights into their mechanical properties [7,8,9,10]. This powerful technique has evolved to provide valuable information about the pericellular layer in the mechanics of the cell body, a crucial component of the cellular microenvironment [11,12,13,14]. The wealth of data generated by AFM has found significant applications in addressing pressing medical challenges, particularly in the realm of cancer detection [15,16]. Numerous studies have demonstrated the utility of nanoindentation AFM in distinguishing between normal and cancerous cells, as well as identifying cells with various metastatic potentials [11,12,17] and even cancer-initiating stem cells [18]. Besides cell mechanics, these distinctions are often based on differences in mechanical properties and the characteristics of the pericellular coat [11,12,19].

However, the application of these findings in clinical settings faces several hurdles because the cells studied are still alive. It is known that the physical properties of living cells are highly sensitive to the environment, particularly during sample preparation. For example, Young’s modulus of cells showed a strong dependence on the number of passages of human breast epithelial cancer cells in vitro [20]. The observed difference was comparable to that between cancer and normal cells reported for human lung cells (~70%) [16]. Additionally, a potential biohazard risk associated with handling living cells in pathology laboratories, as well as additional labor compared to the case with fixed cells, presents serious limitations.

To address these limitations, researchers have proposed the use of AFM to study fixed cells [21]. A special fixation method to preserve the delicate structure of the cell surface was developed [21,22]. This approach has been successfully demonstrated using human cervical epithelial cells [21,23], where AFM imaging of fixed cells has shown promise in detecting various stages of cancer progression. The AFM images were characterized using so-called surface parameters, characteristics of a sample surface previously used in engineering [24]. Initial studies were focused on single surface parameters, such as fractal dimension and multi-fractality, calculated from maps of (nonspecific) adhesion between the AFM probe and sample surface. These parameters proved effective not only in distinguishing between normal and cancerous cells but also in identifying cancer development, from the stages of infection of normal cells with papillomavirus through nonmalignant immortal cells to malignant cells [21]. Nevertheless, utilizing single surface parameters, the accuracy of identification of the stage of cancer development was substantially lower compared to just segregating normal and cancerous cells (74% vs. 100%) [25].

Combining different surface parameters with the help of machine learning (ML) has shown its power. It was reported that by combining five surface parameters, the accuracies of separation of precancerous and cancerous human epithelial cells increased from an area under the curve (AUC), accuracy, sensitivity, and specificity of 0.79, 74%, 58%, and 84% to values of 0.93, 83%, 92%, and 78%, respectively [26]. It was also shown that combining the surface parameters from multiple AFM channels (in addition to the adhesion channel) increased the accuracy substantially in the case of colorectal cells [27].

Recent investigations using exfoliated bladder cells from urine samples further highlighted the potential clinical utility of the developed method. This advanced analytical approach has shown remarkable success, achieving an accuracy of 94% in detecting active bladder cancer when analyzing adhesion maps of just five cells extracted from urine samples (the accuracy dropped to 80% when analyzing just one cell). However, this promising result has been demonstrated for a rather small number of patients. As a result, the statistical significance of that result was relatively modest (the confidence level was only at the minimum acceptable level of 0.05).

In the present work, we validated the ability of the AFM technique to identify human bladder epithelial cancer cells with high accuracy (93% at the level of a single cell) and strong statistical significance (*p* < 0.0001). We further calculated the complete statistical characteristics of the obtained cell classification and determined that the obtained results are statistically significant. This was achieved by using genetically purified human bladder epithelial nonmalignant and cancerous cell lines, which excluded the ambiguity of samples directly obtained from humans due to intrinsic human variability and the fact that some cells can still be nonmalignant even if collected from cancer patients. The use of genetically purified cells minimizes biological variability significantly, enhancing accuracy and reproducibility [28]. In addition to the previous AFM study of bladder cancer, we used a recently introduced AFM modality, Ringing mode [29], which allows the simultaneous collecting of more than ten images/channels of the distribution of different physical properties of the sample surface. In this work, together with the standard height and adhesion channels, we recorded three additional imaging channels, ringing mode (RM) restored adhesion, RM adhesion, and RM viscoelastic adhesion. These five channels are simultaneously recorded to provide multidimensional images of the cell surface. These results add rigor to the ability of AFM to be used to identify active bladder cancer, which may be the first clinical application of AFM.

## 2. Results and Discussion

**Figure 1:**
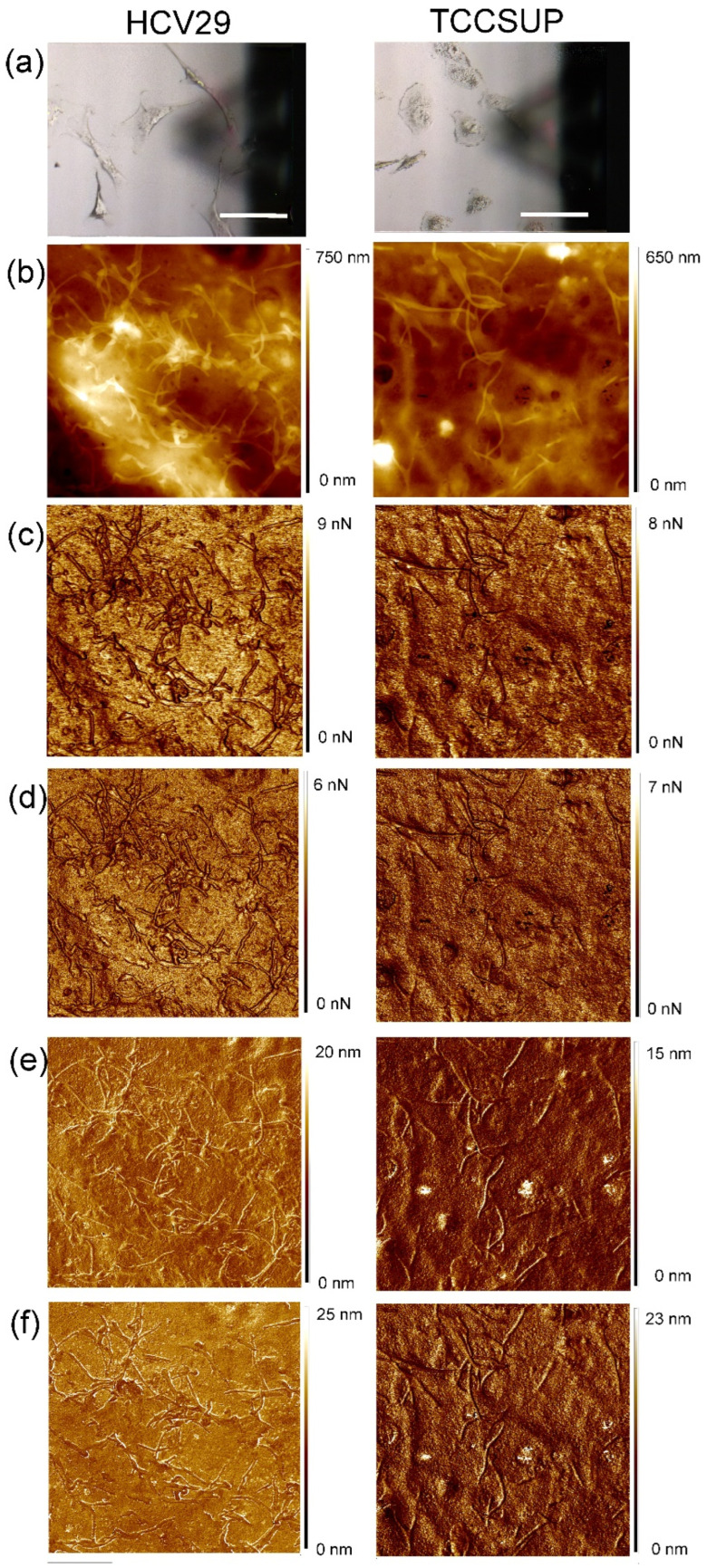
Representative examples of HCV29 and TCCSUP cells. (**a**) Optical images of the cells; the scale bar is 100 µm (one can also see a triangle shadow of the AFM cantilever). Shown are 10 × 10 µm^2^ AFM images recorded in five channels used for the cell classification. (**b**) Height (Nanoscope channel), (**c**) adhesion (Nanoscope channel), (**d**) restored adhesion (ringing mode channel), (**e**) neck size (ringing mode channel), (**f**) disconnection distance (ringing mode channel).

A total of 41 non-cancerous HCV29 cells and 39 malignant TCCSUP cells were imaged by AFM working in ringing mode. Figure 1 shows an example of five channels used for the cell classification for representative HCV29 and TCCSUP cells. There are no clear differences that would allow the identification of malignant cells and non-cancerous cells by using just the human eye.

AFM images carry an enormous amount of information. If one considers each pixel as a single data point/feature, each image of 512 × 512 pixels would be associated with 262,144 features. Following the rule of ten [30], the number of instances (measurements) should be about 10 times larger than the number of features to build a machine learning classifier. Although it is not a strict rule but rather a guidance as it depends on the specifics of data, the needed number of measurements would be completely impractical for AFM. Therefore, we elected the method of reduction of the dimension of the data space [31]. Each image is considered as a surface and processed to obtain so-called surface parameters, the functions of the surface that are used in engineering to characterize surfaces [32]. These parameters include well-known roughness, directionality, fractal properties, etc.; see the full list in the Section 4.

**Figure 2:**
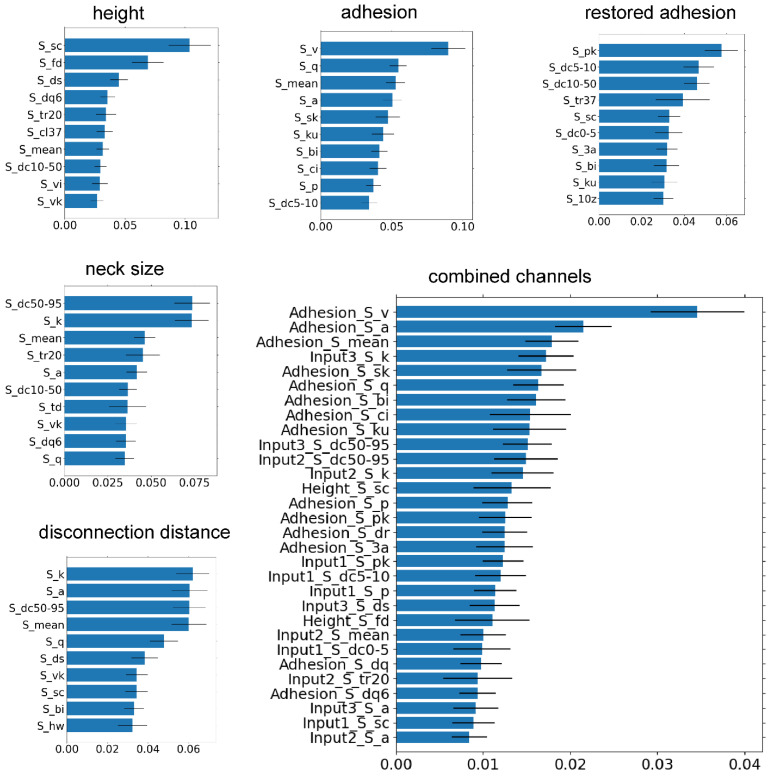
Ranking of surface parameters by Gini importance index. The height (Nanoscope channel), adhesion (Nanoscope channel), restored adhesion (ringing mode channel), neck size (ringing mode channel), and disconnection distance (ringing mode channel) channels, as well as the combined channels, are shown.

The total number of surface parameters is around 37 for each channel. It is still quite large. A further reduction is done by using the Gini importance index analysis. This index ranks the parameters with respect to their importance for cell classification [33]. For future analysis, we keep the 10 highest-ranked surface parameters for each channel and 30 for the combined channels. These parameters and the corresponding Gini index are shown in Figure 2.

**Figure 3:**
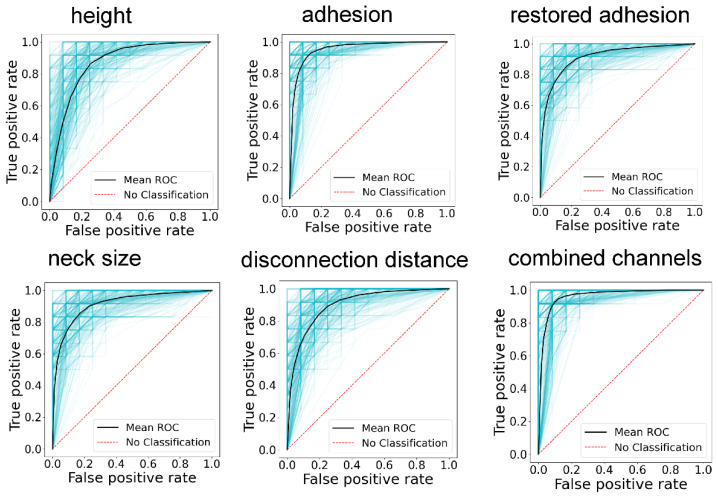
ROC curves described in the classification of nonmalignant and cancerous cells when using the height (Nanoscope channel), adhesion (Nanoscope channel), restored adhesion (ringing mode channel), neck size (ringing mode channel), and disconnection distance (ringing mode channel) channels, as well as the combined channels.

The Random Forest Classifier [34,35] was trained on 70% of the data randomly chosen from the main data set. The statistical results were derived by applying the trained classifier to the remaining 30% of the testing data set. The most comprehensive description of the classifier is the receiver operating characteristic (ROC) curve [36]. Figure 3 shows ROC curves for five chosen channels as well as the combined channel. If these curves cluster around the diagonal, the classifier has no classification power. In other words, the accuracy of such an algorithm is close to none. One can see that all ROC curves are far from the diagonal. Quantitatively, it is better to describe the area under the curve (AUC). It is an integral characteristic of the classifier. If this area is equal to 1, the classifier is 100% accurate. If AUC = 0.5, then the classifier has zero classification power.

The other characteristic of the classifier is the confusion matrix, a 2 × 2 matrix that shows the number of correctly and incorrectly identified cells of both classes [37]. Assuming that the goal is to identify cancerous cells, this matrix consists of four elements: true positive (TP; the percentage of correctly identified cancer cells), false negative (FN; the percentage of cancerous cells falsely identified as nonmalignant), false positive (FP; the percentage of nonmalignant cells falsely identified as cancerous), and true negative (TN; the percentage of correctly identified nonmalignant cells). Based on these four values, one can extract a lot of useful information typically used to characterize various accuracies of the classifier. Specifically, we calculate accuracy, sensitivity, specificity, positive predictive value (PPV), and negative predictive value (NPV). All obtained values for all channels, as well as for combined channels, are summarized in Table 1. One can see that all these characteristics are close to a perfect classifier/predictor (AUC is close to 1).

The statistical significance of the obtained results, specifically the ROC curves, was estimated using the method suggested in reference [31]. Specifically, we calculated the statistical significance for the difference between the array of AUC obtained for the training subset and the same array obtained for the same subset but with randomly assigned classes. The latter case represents the case of no classification power because of the randomness of the class assignment. Both arrays of AUC were obtained using K-fold cross-validation [38]. Because the random assignment of classes can be coincidentally correct for several validation cases, it is paramount to do the K-fold cross-validation several hundred times.

**Figure 4:**
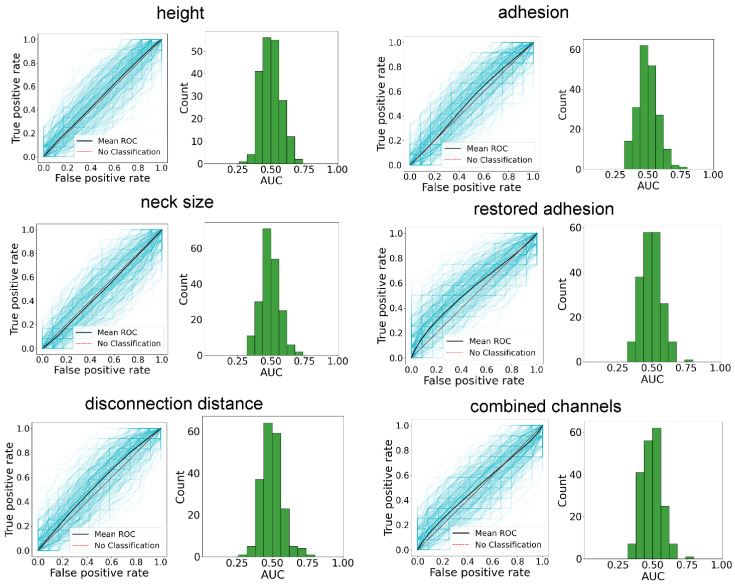
Multiple ROC curves and histograms of the AUCs obtained when the ML algorithm was developed on and applied to the data set with randomly assigned class to each cell. “No Classification” lines are drawn in the graphs showing ROC curves.

Figure 4 shows both multiple ROC curves and histograms of the AUCs obtained when the trained classifier was applied to a testing subset with randomly assigned classes. These ROC curves were obtained for the subsets and algorithms developed on the same data set but with randomized class assignments to each cell. This was done separately for each channel as well as the combined channels. One can see a clustering of the ROC curves, with the AUCs ranging between 0.5 and 0.7. This is significantly smaller than the AUCs of 0.91–0.99 obtained in the original data set. The statistical significance of the difference between these two results was found using the standard one-way ANOVA. We found that the statistical significance was extremely high, *p* < 0.0001 for all channels.

It is instructional to comment on the particular choice of Bruker native and ringing mode channels for the classification of cells. We used two Bruker and three ringing mode channels. The Bruker channels were chosen as being the most robust and weakly dependent on the imaging parameters. The other channels available within the PeakForce QNM suite by Bruker are less suitable for machine learning; see reference [39] for details. Briefly, the deformation channel is very hard to measure precisely because the deformations are small and, thus, strongly dependent on to the sensitivity calibration of the photodetector. The latter tends to drift and require frequent recalibration. The Young modulus channel is a derivative of the deformation; in addition, it is calculated using ad hoc model and taking a very approximate location of the contact point. The peak force error channel is a function of the microscope feedback. It is very sensitive to the peak force, gain parameters (though mainly the integral one), humidity, probe radius, and chosen filters. Moreover, it even depends on the software version of the same microscope. The energy loss channel (the area of the hysteresis between the approach and retraction curve) requires a very precise measurement of the baseline curve to define the point of merging the approach and retraction curves. It also may depend on the software version because of the possible use of filters to improve the detection of the area of the hysteresis.

The three ringing mode channels were chosen because of the following reasoning (only three out of eight available channels were possible to record in the previous version of ringing mode available at the time of the measurements). The restored adhesion channel has the highest signal-to-noise ratio among all ringing mode channels [29]. The neck size channel (shows the physical size of the neck before the probe disconnects from the cell membrane) presents the different mechanical adhesive properties of the cell membrane, which are expected to be specific to cancer [40,41]. The disconnection distance channel presents the size of glycocalyx molecules proved from the cell surface by the action of the AFM probe. It has been demonstrated that glycocalyx changes substantially when cells become malignant [42,43,44].

It is also worth noting that a single channel of adhesion provides quite a high accuracy, 91%, in identifying cancer cells, as shown in Table 1. This naturally raises the question of the necessity of combining the five channels examined in this study. Moreover, three additional channels were derived from the relatively new ringing mode, which is not broadly available. Therefore, it is worthwhile elaborating the advantages of combining AFM channels, particularly those from the ringing mode. In this work, the combination of all five channels yielded only a 2% improvement. However, this was not always the case. For instance, a somewhat similar approach applied to human colorectal epithelial cancer cells of different degrees of aggressiveness demonstrated that the Bruker adhesion channel alone differentiates these cells with only 79% accuracy, while combining it with ringing mode channels increases the accuracy to 94% [27]. Furthermore, there is a significant benefit to combining multiple channels: a substantial decrease in the variability (standard deviation) of the results. One standard deviation of the area under the ROC curve (the most comprehensive statistical parameter) is 3% when using only the Bruker adhesion channel. In contrast, the standard deviation of the area under the ROC curve of combined channels is considerably smaller, only 1%. This indicates that the combined channels provide a more robust outcome. Lastly, it is important to note that every single percentage point above 90% represents a substantial improvement in any clinical method.

Lastly, a more traditional approach based on the detection of biochemical cues of bladder cancer has to be mentioned. It includes more traditional genetic tests and liquid biopsy tests looking for specific chemical biomarkers. FDA-approved biomarkers such as bladder tumor antigen (BTA) STAT and TRAK detect complement factor H-related proteins in urine, with sensitivities ranging from 56–83% and specificities up to 93%; however, false positives are still a problem. It occurs in benign conditions like inflammation or hematuria [45,46]. Nuclear matrix protein 22 (NMP22), which measures nuclear proteins released during tumor apoptosis, has shown sensitivities of 74–100% and specificities of 55–90%, making it useful for detecting bladder cancer recurrence but not as a standalone diagnostic tool, mainly because of such high variability of the accuracy across the board [46,47,48]. Genetic mutations such as FGFR3 are associated with low-grade tumors, while TP53 alterations are linked to high-grade invasive cancers, reflecting distinct molecular pathways in bladder cancer progression. Epigenetic markers like RASSF1A methylation correlate with advanced tumor stage and poor prognosis, suggesting their potential as prognostic indicators [49]. Protein-based markers such as survivin, an inhibitor of apoptosis, have been detected in urine samples of bladder cancer patients with high specificity and correlate with tumor recurrence [50,51]. Extracellular vesicles and non-coding RNA are growing areas in the search for novel urinary biomarkers [52].

While these biomarkers improve diagnostic accuracy and reduce reliance on invasive procedures like cystoscopy, challenges remain due to high variability in their performance and the need for further clinical validation [3,52,53]. Because our approach is based entirely on the biophysical properties of cells, it is expected to be complementary to the biochemical approach. Additional research is needed to understand the possible correlations and complementarity of these two approaches.

## 3. Conclusions

This study strengthens the scientific foundation for AFM-based detection of bladder cancer cells by demonstrating its effectiveness in a controlled system using genetically purified cell lines. Our approach, combining machine learning algorithms with AFM adhesion maps, achieved highly accurate cancer cell identification within this well-defined model system. The implementation of ringing mode AFM, enabling simultaneous acquisition of multiple physical property channels, further enhanced detection capabilities with exceptional statistical significance (*p* < 0.000001). While these findings corroborate the potential of AFM-based cancer detection shown in previous clinical studies, it is important to note that our use of a single cell line model, though methodologically rigorous, does not capture the full spectrum of inter-individual variability present in clinical settings. Thus, these results provide strong mechanistic support for the underlying principles of AFM-based cancer detection but not yet full clinical validation. Given the pressing need for alternatives to invasive and costly cystoscopy, our findings represent an important technical foundation for this promising diagnostic approach. Future research should focus on bridging the gap between these controlled laboratory findings and clinical applications by conducting larger-scale patient studies that account for human biological variation. Future increases in the speed of AFM imaging and operator-independent scanning will make this AFM-based approach a promising contributor to the area of oncological diagnostics.

## 4. Experimental Section

*Cells:* The present study employed two distinct bladder cell lines: non-cancerous ureteral cells (HCV29, established from the Institute of Experimental Therapy, Polish Academy of Sciences in Wroclaw, Poland) and malignant transitional cell carcinoma cells (TCCSUP, procured from the ATCC via LGC Standards). The HCV29 cell culture was performed in an RPMI-1640 medium enhanced with 10% fetal bovine serum (FBS). TCCSUP cells were maintained in EMEM with identical FBS supplementation. The choice of culture medium reflects the specific physiological requirements of these cells. Both cell lines were cultured in standard culture flasks (Sarstedt, Nümbrecht, Germany) under controlled conditions maintained by a Nuaire incubator, which provided a consistent environment of 37 °C, 5% CO_2_, 95% air, and >98% relative humidity. Cell passaging was initiated upon reaching 80–90% confluence, utilizing differentiated trypsinization protocols: HCV29 cells were treated with 0.05% trypsin-EDTA, whereas TCCSUP cells required 0.25% trypsin-EDTA solution, both with 4-min exposure times. Following several passages, cells between passages 6 and 8 were used. The cells were cultured on glass slides.

*Cell fixation protocol:* The cells were fixed using Karnovsky fixative [21,22]. The cells were washed twice with prechilled (to 4 °C) phosphate-buffered saline (PBS, Sigma-Aldrich, Burlington, MA, USA) and immersed in prechilled (to 4 °C) Karnovsky fixative (Fisher Scientific, Waltham, MA, USA). The fixation was processed overnight at 4 °C. Note that Karnovsky fixative should be handled under a fume hood. Afterward, the glass slides with cells were rewashed twice, immersed in PBS, and shipped to Tufts University using lower-temperature packaging. After receipt at Tufts University, the samples underwent PBS washing, followed by an overnight desalination in deionized water at 4 °C. The preparation concluded with a freeze-drying process, utilizing a standard Labconco freeze-dryer (Kansas City, MO, USA) operating at −55 °C for 30–45 min. The slides with cells were taken out from the freeze dryer when the temperature of the slides reached about the dew point temperature.

Atomic force microscopy: Surface imaging of the cells was performed using an Icon atomic force microscope equipped with a Nanoscope V controller (Bruker, Inc., Santa Barbara, CA, USA) and ringing mode extension (NanoScience Solutions, Inc., Arlington, VA, USA). Bruker ScanAsyst Air cantilevers with a nominal spring constant of 0.4 N/m were used for imaging, with the exact spring constant determined through the thermal tuning option in the Nanoscope software (http://nanoscaleworld.bruker-axs.com/nanoscaleworld/ accessed on 12 December 2024). The photodetector sensitivity was calibrated against clean glass surfaces. The curvature radius of the cantilever tips was approximately 3 nm (identified using tip check samples). Typically, one AFM probe is sufficient to image dozens of cells without being contaminated. Possible contamination of the AFM probe was identified by an abnormal increase of disconnection distance in the disconnection distance channel (this channel is much more sensitive to probe contamination than the adhesion channel that has usually been used for these purposes before). Images of cell surfaces measuring 10 × 10 µm^2^ were captured at a resolution of 512 × 512 pixels, with a scanning speed of 0.4 Hz to ensure that surface parameter extraction remained independent of scanning speed. Imaging each cell at this speed took approximately 18 min. All scans were conducted at room temperature, with humidity levels maintained below 70%, as higher humidity can affect image quality [25]. One 10 × 10 µm^2^ area to scan per cell on the cell surface is chosen randomly but preferably on relatively smooth areas of cells by trying to avoid the areas of the big change of topography because of the different geometry of the tip contact. These areas can easily be identified with the help of an optical microscope attached to the AFM.

**Figure 5:**
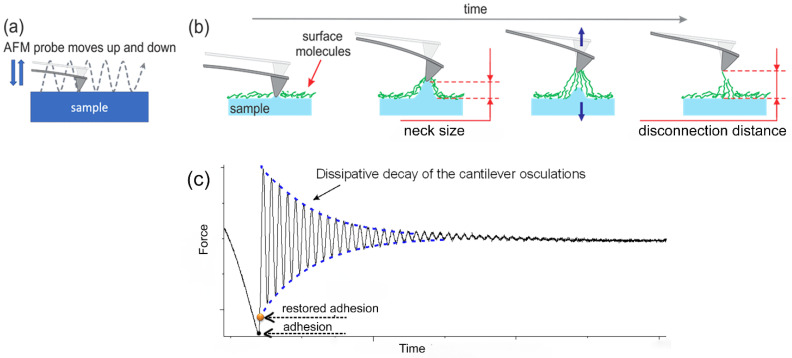
Graphical description of the ringing mode channels used in this work. (**a**) Trajectory of the AFM probe scanning in subresonance tapping. (**b**) Graphical presentation of the neck size and disconnection distance channels. (**c**) Explanation of the restored adhesion.

*Ringing mode channels used in this work:* For the classification of cells, the following five imaging channels were used: height (Nanoscope channel), adhesion (Nanoscope channel), restored adhesion (ringing mode channel), neck size (ringing mode channel), disconnection distance (ringing mode channel). A detailed description of these channels can be found in references [54,55] Here, we just briefly describe these channels. Figure 5 shows the basics of imaging in a subresonance tapping, which is common for both PeakForce QNM and ringing mode. In this mode, the AFM probe moves vertically relative to the sample in a sinusoidal motion, gently tapping into the surface of a sample. At the same time, the probe moves in the lateral direction about the sample surface (Figure 5a). During each tap, the AFM probe develops some adhesion with the surface and eventually disconnects from the surface. Figure 5b shows the process of disconnection in time. At the beginning of the disconnection, the probe may pull off a neck from the cell surface. Neck size is the height of the neck right before disconnection with AFM probe. It is measured and presented in the neck size channel. If the sample surface is covered with molecules that can stick to the AFM probe, the probe pulls up those molecules before they disconnect from the probe. This disconnection distance is measured and presented in the disconnection distance channel.

The restored adhesion can be understood as follows. During disconnection of an AFM probe from the sample surface, the AFM cantilever loses energy. Right after the disconnection, the AFM cantilever starts to oscillate (ring) following the equations of the damping harmonic oscillator. Extrapolating this oscillating amplitude to the past, to the moment of the disconnection of the AFM probe from the sample surface, one can obtain the value of the deflection of the cantilever, or force at that moment. This is called “restored adhesion”. If there were no energy losses, the restored adhesion would be equal to the adhesion force. In reality, the restored adhesion is always lower than the value of the adhesion force; see Figure 5c. The advantage of the restored adhesion is in its lower signal-to-noise ratio compared to other channels, including the adhesion channel. This is because the restored adhesion is calculated using multiple points of the ringing curve (in contrast, the adhesion is just one point, which is quite challenging to identify due to the finite digitalization and analysis of the deflection curve of the cantilever).

*Preparation of data for machine learning. Service parameters:* Raw data of AFM images were utilized directly, with the height channel data being the sole exception requiring plain fitting. Then the images were converted into the surface parameters. The used surface parameters are the functions of the images, which are routinely used in multiple engineering applications to characterize surfaces [56]. We used the software MountainsSPIP (latest v. 9.2, by Digital Surf, France), which automatically calculates the surface parameters for 3D image surface arrays recorded by AFM. In this study, we applied it for all five channels (AFM images are digital arrays of either heights or adhesion). A complete list of surface parameters used in the present work is as follows: roughness average (S_a), root mean square (RMS) (S_q), surface skewness (S_sk), surface kurtosis (S_ku), peak-peak (S_z), ten point height (S_10z), max valley depth (S_v), max peak height (S_p), mean value (S_mean), mean summit curvature (S_sc), texture index (S_tdi), root mean square gradient (S_dq), area root mean square slope (S_dq6), surface area ratio (S_dr), projected area (S_2a), surface area (S_3a), surface bearing index (S_bi), core fluid retention index, (S_ci), valley fluid retention index (S_vi), reduced summit height (S_pk), core roughness depth (S_k), reduced valley depth (S_vk), l-h% height intervals of bearing curve (S_dc), density of summits (S_ds), texture direction (S_td), texture direction index (S_tdi), dominant radial wavelength (S_rw), radial wave index (S_rwi), mean half wavelength (S_hw), fractal dimension (S_fd), correlation length at 20% (S_cl20), correlation length at 37% (S_cl37), texture aspect ratio at 20% (S_tr20), and texture aspect ratio at 37% (S_tr37). We considered each channel/image independently for cell classification. This approach gives us information about the contribution of each channel in the identification of cell phenotype, while combining the channels brings higher accuracy in the identification of cancer cells.

Since we take only one 10 × 10 µm^2^ image of the cell surface, it is advisable to verify possible variations of this image of the cell surface. To do that, we use the following simple test by splitting 10 × 10 µm^2^ images into four 5 × 5 µm^2^ quadrants. The surface parameters can be calculated and compared separately for each quadrant. The difference between these parameters should be minimal, although it might be difficult to understand if the difference is large or small from the point of view of the desired classification of cells. Therefore, the following simple machine learning test can be performed. The classification can be done using the surface parameter average for each cell by all four quadrants and then comparing the same when the surface parameters for each cell are calculated as the median value for the quadrants. The results reported in this work stay the same for both of these methods of calculating the surface parameters per cell.

*Machine learning classifier algorithms:* The Gini index *G* was used to keep the most important surface parameters (variables) for the classification. The Gini index is a measure used to evaluate the importance of features in decision trees. The Gini importance measures how much each feature contributes to decreasing impurity across all nodes in a decision tree; see reference [33] for details. Random Forest Classifier [57,58,59,60,61,62,63,64] was used for the analysis of images of nonmalignant and cancerous cells. The Pandas Python package (version 0.18.1). and scikit-learn Python machine learning package (version 0.17.1) were used. The following parameters were used for the classifier: max_depth = 10, n_estimators = 100, bootstrap = false. To split the database for testing and training subsets, we used 70% of the data for training and 30% for testing. This splitting was randomly repeated 500 times (K-cross validation) to avoid the coincidental choice of particular data and to demonstrate the robustness of the obtained results. The split data were stratified to keep the cells of both classes in equal representation. Training of the classifier does not take a long time. Even with several hundred K-cross validations, it takes less than half an hour on a rather modest i7 intel Windows computer.

*Statistical analysis:* The performance of the machine learning classifier was evaluated through ROC curves and confusion matrices, with distributions represented by ROC curve clusters and area-under-curve histograms. We also calculated the accuracy, sensitivity, specificity, positive predictive value (PPV), and negative predictive value (NPV) of the method. The statistical significance of the obtained results was estimated using the method suggested in reference [31]. Specifically, the array of AUCs obtained during K-cross validation of the classifier applied to the training subset was compared to the same array of AUCs obtained for the training subset that had a randomly assigned class (either nonmalignant or cancerous). Because the latter can be coincidentally correct, it is paramount to do the K-cross validation several hundreds of times. The statistical significance of the difference between these two arrays is found using standard one-way ANOVA.

## Figures and Tables

**Table 1:** Full characteristic of classification of nonmalignant and cancerous cells. Area under the ROC curve, parameters of the confusion matrix, accuracy, sensitivity, specificity, positive predictive value (PPV), and negative predictive value (NPV) are shown. *P = TP + FN, N = FP + TN*.

		Height	Adhesion	Restored Adhesion	Neck Size	Disconnection Distance	Combined Channels
**AUC**	*Area under the ROC curve*	0.91	0.97	0.95	0.94	0.93	0.99
**TN**	True negative	80%	91%	90%	86%	84%	93%
**FP**	False positive	20%	9%	10%	14%	16%	7%
**FN**	False negative	13%	10%	14%	14%	17%	7%
**TP**	*True positive*	87%	90%	86%	86%	83%	93%
**Accuracy**	$\frac{TP+TN}{P+N}$	83%	91%	88%	85%	83%	93%
**Sensitivity**	TP/P	87%	90%	86%	85%	82%	93%
**Specificity**	TN/N	80%	91%	90%	86%	84%	92%
**PPV**	$\frac{TP}{TP+FP}$	81%	91%	90%	86%	84%	93%
**NPV**	$\frac{TN}{FN+TN}$	86%	90%	86%	85%	83%	93%

## Data Availability

The original contributions presented in this study are included in the article/Supplementary Materials. Further inquiries can be directed to the corresponding author.

## Supplementary Materials

The following supporting information can be downloaded at: https://www.mdpi.com/article/10.3390/cells14010014/s1, Surface parameters used to obtain the results reported in the paper.
